# Recent research progress on the synthesis and biological effects of selenium nanoparticles

**DOI:** 10.3389/fnut.2023.1183487

**Published:** 2023-05-16

**Authors:** Ting Zhang, Meng Qi, Qian Wu, Peng Xiang, Dejian Tang, Qiang Li

**Affiliations:** ^1^Key Laboratory of Coarse Cereal Processing, Ministry of Agriculture and Rural Affairs, Sichuan Engineering and Technology Research Center of Coarse Cereal Industrialization, School of Food and Biological Engineering, Chengdu University, Chengdu, Sichuan, China; ^2^Ankang R&D Center for Se-enriched Products, Ankang, Shaanxi, China; ^3^Key Laboratory of Se-enriched Products Development and Quality Control, Ministry of Agriculture and Rural Affairs, Ankang, Shaanxi, China

**Keywords:** selenium nanoparticles, biological activity, synthesis, agriculture, food

## Abstract

Selenium is an essential trace element for the human body, with the chemical and physical characteristics of both metals and nonmetals. Selenium has bioactivities related to the immune system, antioxidation, anti-virus, and anti-cancer. At the same time, it also plays a role in reducing and alleviating the toxicity of heavy metals. Compared with inorganic selenium, organic selenium is less toxic and has greater bioavailability. Selenium nanoparticles (SeNPs) have the advantages of high absorption rate, high biological activity, and low toxicity, and can be directly absorbed by the human body and converted to organic selenium. Selenium nanoparticles have gradually replaced the traditional selenium supplement and has broad prospects in the food and medical industries. In this paper, the chemical, physical, and biological methods for the synthesis of selenium nanoparticles are reviewed, and the microbial synthesis methods of selenium nanoparticles, the effects of selenium nanoparticles on crop growth, and the antibacterial, antioxidant, anticancer, and anti-tumor effects of selenium nanoparticles are also systematically summarized. In addition, we evaluate the application of selenium nanoparticles in selenium nutrition enhancement, providing support for the application of selenium nanoparticles in animals, plants, and humans.

## 1. Introduction

Selenium, a nonmetal and an essential micronutrient for humans and animals, plays an important role in the human body by improving the activities of glutathione, peroxidase, selenidase, and other enzymes, which can protect the human body from immune-related diseases (1). The normal selenium level of adults is about 81 mg; the daily dietary requirement is about 55 mg; and the upper limit is about 400 mg (2). The bioavailability, toxicity, and antioxidation of selenium depend on its chemical form. In the environment, selenium usually exists in an inorganic form, mainly as selenite and selenate, but organic forms can also be found (i.e., selenium amino acids, methylated compounds, selenoproteins, and derivatives) (3). Selenium deficiency in the body is related to the increased risk of many diseases. Selenium deficiency affects the synthesis of selenoprotein and causes a reduction in glutathione peroxidase (GPx) activity, leading to a reduction in the ability of tissue cells to resist oxidative damage. This directly affects cell division, reproduction, immunity, and the development of metabolic disorders, thus interfering with the synthesis and metabolism of nucleic acids, proteins, mucopolysaccharides, and enzymes (4). Selenium deficiency causes various diseases, such as thyroid diseases, diabetes, human reproductive disorders, and obesity. Therefore, selenium supplementation is also very important. However, due to the small gap between the effective dose and the toxic dose, the range of safe concentration is narrow, the absorbed amount is difficult to control, and excess levels are harmful to the human body (5). Selenium nanoparticles (SeNPs), a new form of selenium supplement, is essentially inorganic selenium, but because of its size, it has special physical and chemical properties, strong activity, low toxicity, good absorption, and improved safety, compared to inorganic and organic selenium. SeNPs is in a highly stable colloidal state and has demonstrated several biological activities, such as immune system, anti-oxidation, anti-virus, and anti-cancer activities, in addition to being synthesized for nutritional fortification and developed for medical applications (6, 7).

At present, physical, chemical, and biological preparation methods are mainly used to synthesize SeNPs. Due to their unique properties, nanoparticles play a vital role in various fields, including biomedical, environmental, and agricultural industries. SeNPs have been proven to be a potential material to alleviate several problems caused by the formation of biofilm, ROS production, and low redox activity (8). Compared with organic selenium and inorganic selenium compounds such as sodium selenite and sodium selenite, selenium nanoparticles are used in food safety applications (9–11) as antibacterial nano-coating, food packaging and functional food because of its high biological activity, high bioavailability, low toxicity, particle dispersion and large surface area (12). And marketed as a food additive to tea products with a variety of health benefits (13). Selenium can also be used in fertilizer to promote plant growth, increase crop yield, and promote the development of selenium bio-enhanced crops. However, SeNPs are not stable and tend to aggregate; therefore, it is very necessary to develop simple and efficient methods to increase the dispersion and stability of selenium nanoparticles (14).

In recent years, the number of studies on SeNPs has increased. Khurana et al. (15) introduced the role of SeNPs for pharmacological protection against various inflammatory and oxidative stress-mediated conditions. Thereafter, the synthesis and characterization of SeNPs were also reviewed, with an emphasis on their role and application in human health and the environment (16). Ullah et al. (17) summarized the application of SeNPs (from probiotics/non-pathogenic organisms) as a promising anticancer drug, and selenium nanoparticles from probiotics/non-pathogenic organisms is considered safe for human consumption. Bozena et al. (18) reviewed the biological effects of SeNPs in organisms, their advantages, absorption mechanism, and the application of nanotechnology in oral drug delivery. However, at present, there is no systematic summary of the synthesis methods of SeNPs and the effects of SeNPs on crops, humans, and animals. This limits our comprehensive understanding of SeNPs preparation and the biological effects of SeNPs. In this paper, the preparation methods of SeNPs are comprehensively and systematically reviewed, and the advantages and disadvantages of each preparation method are compared (Table 1). The biosynthesis methods of SeNPs are emphatically introduced. The effects of SeNPs on soil, crops, animals, and the human body are also discussed. The antioxidation, anti-bacterial, anti-cancer, and anti-tumor effects of SeNPs are summarized, with the view of providing more valuable references for selenium SeNPs.

**Table 1:** Comparison of synthesis methods of SeNPs.

	Method	References	Advantage	Shortcoming
	Hydrothermal method	(19)	Low process temperature, low energy consumption and environmental friendliness	
				High equipment cost and poor stability
Physical method	Electrochemical method	(20)	High selectivity and green.	
	Liquid phase pulsed laser ablation	(21)	Low equipment cost, easy to collect SeNPs and the stable storage SeNPs in colloidal solution.	
	Photocatalysis	(22)	High preparation efficiency and reusable.	High energy consumption, accompanied by the production of irritant chemicals, the synthesized SeNPs has poor stability and is easy to be converted into gray-black elemental selenium, which then increases toxicity and loses activity.
Chemical method	Ascorbic acid reduction method		Low cost, convenient source of raw materials, high activity, small particle size, high temperature resistance, strong stability and uniform size of SeNPs.	The conditions for the synthesis of SeNPs by microbial method are relatively harsh, and the culture conditions such as pH and temperature need to be strictly controlled
Biological method	Microbial reduction method		Simple process, it is environmentally friendly and uses reductants that are easily accessible and biodegradable.	

	Plant extract synthesis method	(23)		

## 2. Preparation of selenium nanoparticles

The synthesis of SeNPs is achieved by chemical, physical, and biological methods. The chemical synthesis method involves the reduction of high-valence selenium to a simple state in a reduction reaction system. Over the past 30 years, chemical synthesis has been considered the gold standard for the synthesis of SeNPs particles, including catalytic reduction, precipitation, and decomposition methods. However, the use of highly toxic chemicals and the generation of chemical pollution caused by these reactions have limited their large-scale production and use (24, 25). Physical synthesis is carried out by exciting and releasing electrons by physical means, such as photocatalysis and pulsed laser cauterization, and the high-valence selenium source forms stable elemental selenium after receiving electrons. Biosynthesis is a rapidly developing, environmentally-friendly method for the synthesis of SeNPs. The biosynthesis of SeNPs from plant extracts, fungi, algae, and bacteria has attracted more and more attention (26). The shape, size, and degree of aggregation of SeNPs affect the biological activity of SeNPs. Therefore, it is particularly important to synthesize green and effective SeNPs with high biological activity.

### 2.1. Physical synthesis

The most commonly used physical methods for the synthesis of SeNPs include hydrothermal, microwave radiation, laser cauterization, electrochemical, and so on (27). Compared to other traditional methods, such as the wet chemical route, physical vapor deposition, and chemical vapor deposition for the synthesis of selenium, pulsed-laser liquid ablation in liquids (PLAL) has several advantages, including a simple structure, a high surface purity of the SeNPs (with no unnecessary adducts or byproducts) (28), and easy collection or storage of the resulting nanoparticles as colloidal solutions. In addition, a vacuum room or clean room environment is not required, making it a simple and green technology (29). However, the nanoparticles synthesized by the liquid phase pulsed laser ablation (LP-PLA) method may not be contaminated by chemical reagents. Because there is no other vacuum chamber, the equipment cost is low, and it is easy to collect nanoparticles after synthesis, and nanoparticles can be stored stably in colloidal solution (29). Liquid pulsed laser turbidimetry is a new PLAL process for SeNPs that can be used for the preparation of pure SeNPs (without surface functionalization). The SeNPs obtained by twice pulsed laser irradiation have small sizes, high stability, and a variety of biological functions (30).

Overschelde et al. (21) synthesized pure SeNPs in solution with a 248 nm excimer laser. The excimer laser (248 nm) worked at a low flux (F ~ 1 J/c m2) and was used to synthesize a colloidal solution of SeNPs. In addition, Guisbiers et al. (31) synthesized pure SeNPs by immersing 99.999% selenium powder in deionized water and ethanol using a pulsed nanosecond Nd-Y AG laser. After they successfully synthesized the SeNPs, the antibacterial test results showed the SeNPs significantly reduced the number of *Escherichia coli* and *Staphylococcus aureus* cells. In addition, the maximum concentration required to inhibit *E. coli* or *S. aureus* for 24 h was at least 50 ppm. Thus, SeNPs synthesized by PLAL in deionized water were effectively used as antibacterial therapeutic agents. Importantly, the bacterial density was reduced without using antibiotics (32). Shar et al. (19) synthesized nano-sized selenium particles by hydrothermal method, which is simple, environmentally friendly, and inexpensive. Sodium selenite was used as a precursor, and L-ascorbic acid was used as a reducing agent and stabilizing agent. Mellinas et al. (33) used microwave radiation to prepare SeNPs using *Theobroma cacao* L. bean shell extract, which effectively controlled the size of SeNPs, extended their storage time, and enhanced their antioxidant activity.

### 2.2. Chemical synthesis

Chemical reduction of SeNPs usually involves adding stabilizers or dispersants and reducing agents to sodium selenite solution. The stabilizers include polysaccharides, proteins, and surfactants (34). From the perspective of environmental protection, energy conservation, and preparation methods, polysaccharides are more suitable as stabilizers for SeNPs than proteins, polyphenols, and other materials. Polysaccharides have active hydroxyl groups and complex branching structures, which can modify the interface of nanoparticles, control the growth of nanoparticles, and stabilize the nanoparticle solution (35). SeNPswere prepared by adding CVPs into a redox system of selenite and ascorbic acid.

Zhang et al. (36) prepared nano-sized selenium particles by reducing selenite solution with ascorbic acid in the presence of water-soluble polysaccharides, such as chitosan (CTS), konjac glucomannan (KGM), acacia (ACG) and carboxymethyl cellulose (CMC). The monodisperse spherical selenium colloids obtained were very stable in solution and did not agglomerate for at least 6 months. Under certain experimental conditions, SeNPs with nano-rod-like and fractal morphology can be obtained. Chen et al. (37) used chitosan (CS) and carboxymethyl chitosan (CCS) as a stabilizer and cap agents, respectively, to synthesize monodisperse SeNPs by a simple synthesis method.

Although SeNPs has good biological activity and low toxicity, the stability of selenium nanoparticles must be improved. Thus, Zhang and others (38) prepared polysaccharide SeNPswith spirulina polysaccharide as a stabilizer by reacting sodium selenite and ascorbic acid. The prepared SeNPs were spherical, with an average particle size of 73.42 ± 0.69 nm, and they could be stored for 75 days at 4°C.

### 2.3. Biological synthesis

Biosynthesis of SeNPs usually involves adding microorganisms or plant extracts to the sodium selenite solution. The microorganisms use their own metabolism to reduce high-valence selenium to SeNPs and, at the same time, generate organic substances, such as proteins, polysaccharides, and lipids, on the surface of SeNPs to stabilize the structure. Plant extracts are rich in polyhydroxy substances, which can be used as reducing agents, and strong complexing substances (i.e., amino and aldehyde groups), which can be used as stabilizers. The biosynthesized SeNPs has high activity, small particle size, high temperature resistance, strong stability, and uniform size. The use of natural organisms, microorganisms, microalgae, enzymes, plants, and plant extracts provides a reliable, simple, non-toxic, low-cost, and eco-friendly method (39). Therefore, the green synthesis of SeNPs has attracted much attention because it has promoted the development of alternative, sustainable, safer, less toxic, large-scale, and environmentally-friendly methods (40).

The toxic chemicals, high temperature conditions, and expensive equipment required of the physical and chemical synthetic methods make the prohibitive for large-scale production (41). In contrast, the biosynthesis of nanoparticles uses biological organisms, such as plants, microalgae, and other microorganisms with low toxicity to the environment (42). Biological preparation using plants, plant extracts, enzymes, and microorganisms is considered the most environmentally-friendly approach. Therefore, further development of the biosynthesis of SeNPs, to regulate the morphology and particle size, is worthy of exploration.

#### 2.3.1. Microbial reduction method

Biogenic nanoparticles are synthesized by bacteria, fungi, yeast, plants, and other microorganisms inside or outside the cell, and these molecules are used as reducing and stabilizing agents (43). In some bacteria, selenium is a toxic substance that is metabolized, and bacteria have evolved methods to eliminate this toxicity. The most significant is the formation of elemental selenium nanoparticles.

Fesharaki et al. (44) tested the ability of a *Klebsiella pneumoniae* to synthesize SeNPs from selenium chloride. A *Klebsiella pneumoniae* culture solution containing SeNPs was sterilized at 121°C and 17 psi for 20 min. The size of the released SeNPs was between 100 and 550 nm, and the average particle size was 245 nm. This study also showed the hygrothermal sterilization process can successfully recover selenium from bacterial cells. Avendano et al. (45) reported the ability of a previously unrecognized soil bacterium, *Pseudomonas* KT2440, to synthesize SeNPs from selenite. The soil bacterium *P. putida* KT2440 was able to produce SeNPs from selenite using an environmentally friendly process, avoiding the traditional use of chemical products and high temperatures.

Because SeNPs are usually synthesized by anaerobic bacteria and surface soil contains mostly aerobic bacteria, anaerobic bacteria cannot be directly used for bioremediation of contaminated surface soil. Wang et al. (46) identified a selenite reducing bacteria, *Freundii* Y9, which exhibited high selenite reducing energy. Under aerobic and anaerobic conditions, SeNPs were synthesized, and this bacteria can be used for both surface soil and sub-surface soil.

**Figure 1:**
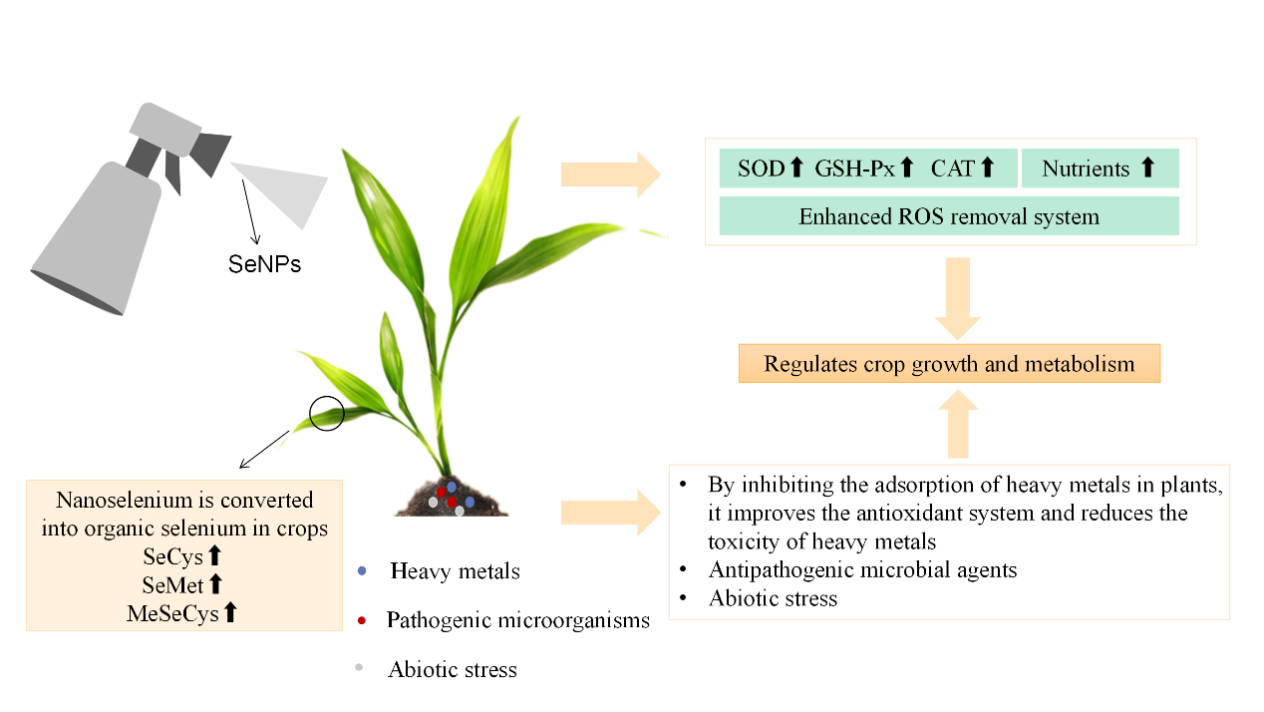
Application and biological effects of SeNPs in crops.

Pouri et al. (47) used *Bacillus cereus* to biosynthesize spherical SeNPs without any environmental pollution and chemical substances. The bacterial cultures were prepared in sodium selenate solution and incubated at 30°C for 24 h. The nanoparticles were centrifuged, washed with 0.9% NaCl, cleaned by ultrasound, washed with sodium dodecyl sulfate (SDS) in ammonium trichloride, and separated by a water–octanol two-phase system. Then, UV–VIS spectroscopy, dynamic light scattering (DLS), scanning electron microscopy (SEM), and X-ray diffraction (XRD) analyses were used to confirm the synthesis of 170 nm diameter spherical SeNPs, without the generation of environmental pollution. In addition, these particles had a high absorption rate, which is conducive to the application of SeNPs in medicine (Figure 1).

Zhang et al. (48) first used the mycelium of *Mariannaea* spp. to carry out the biosynthesis of SeNPs. Tugarova et al. (49) developed an original and simple method to synthesize extracellular SeNPs (SeNPs) of relatively uniform size, using the strains Sp7 and Sp245 of the ubiquitous rhizosphere bacteria *Azospirillum brasilense*. Both strains can reduce selenite (SeO_3_^2−^). In addition, a reliable purification procedure that can be used to recover SeNPs has also been improved. Fischer et al. (50) also found that by changing the conditions for bacteria to reduce selenite, bacteria can be used to obtain extracellular targeted SeNPs. Fischer et al. found aerobic *Bacillus safensis* JG-B5T reduced 70% of 2.5 mM selenite to form red spherical biological selenium nanoparticles (BioSeNPs). Through transmission electron microscopy imaging, biological SeNPs were only produced outside the cell.

In addition, Ullah et al. (51) used *Bacillus subtilis* BSN313 as a raw material to produce SeNPs economically and easily via biosynthesis. The yield of SeNPs reached 5–200 μg/mL, and the average particle size of SeNPs was 530 nm. The zeta potential was −26.9 (mV), the electrophoretic mobility was −2.11 μm cm/Vs, and 200 g/mL SeNPs showed antibacterial activity against *E. coli*, *S. aureus*, and *Pseudomonas aeruginosa.*

The preparation of SeNPs and organic selenium by microorganisms is not only affected by their own selenium tolerance and selenium accumulation ability but also by other external conditions, such as the selenium addition method, culture temperature, pH value, culture medium, oxygen flux, selenium source, and exogenous ions.

#### 2.3.2. Plant extract synthesis method

Green nanotechnology using plant extracts reveals new possibilities for the synthesis of new nanoparticles with ideal characteristics required for the development of biosensors, biomedicine, cosmetics, and nanotechnology. This technology also has potential in electrochemical, catalytic, antibacterial, electronic, sensing, and other applications (52). The functional groups of phytochemicals that induce the synthesis of nanoparticles are amines and alkanes, and these groups widely exist in metabolites, such as flavonoids, tannins, alkaloids, steroids, and terpenes. They are conducive to the biological reduction of metal nanoparticles into elemental forms (53). The reducing agents used are easily available and biodegradable. The plant extract is rich in protein, vitamins, amino acids, and other components, which can be used as reducing agents. At the same time, the SeNPs can be stably synthesized.

El-Sayed et al. (54) successfully synthesized SeNPs using the cultured extract of *Rhodosinum* spp. grown on bagasse in solid state fermentation. The synthesized SeNPs showed a single-phase crystal structure, with a spherical shape and an average particle size of 46.58 nm. Dynamic light scattering analysis showed the synthesized SeNPs was monodisperse. The recorded polydisperse index value was 0.205, and the zeta potential value was −24.01 mV, indicating the SeNPs was highly stable.

Yilmaz et al. (55) synthesized SeNPs using tarrartemisia extract as the reducing agent. The results showed tarrartemisia extract stabilized the dispersion of SeNPs into quasi-spherical nanoparticles, with a particle size of 20–50 nm, and SeNPs existed in the form of nanocrystals. Selenium was embedded in the nanostructures and exhibited high thermal stability (above 200°C). Hashem et al. (56) demonstrated in a prior study that the aqueous extract of nettle leaves was utilized to synthesize SeNPs in an environmentally-friendly way, and the antibacterial and anticancer activities of the resultant SeNP were evaluated. The SeNP showed promising antibacterial activity against Gram-positive and Gram-negative bacteria, as well as single-cell and multi-cellular fungi. The potential antibacterial, antifungal, and anticancer effects of this material bestow a certain application value in the medical field.

However, the plant extraction synthesis method requires a large number of plants, making the process complicated and time-consuming (57). In contrast, the microbial reduction synthesis of selenium nanoparticles is a more efficient method (51).

## 3. Effect and mechanism of selenium nanoparticles on crops

In recent years, SeNP has attracted much attention in agricultural applications, because it can alleviate a variety of biological and abiotic stresses, such as heavy metals, salinity, drought, and high temperature (58). In addition, it can inhibit pathogenic microorganisms in crops. The absorption, transport, and distribution of selenium depend on the crop type, development stage, chemical form, concentration, physiological conditions (salinity and soil pH), the presence of other substances, the activity of membrane transporters, and the transport mechanism of crops (59). Many studies have shown that SeNPs can be used as a fertilizer to improve crop yield. The impact of selenium nanoparticles on different crops varies greatly, due to different crop growth stages, exposure methods, and exposure time. Additionally, it depends on the shape, size, chemical composition, concentration, surface structure, aggregation, and solubility of SeNPs (60). Recent studies have shown selenium (Se) can alleviate various abiotic stresses in applied crops (61). Selenium mainly uses sulfur (S) transport and the S metabolism pathway. Se helps eliminate free radicals and ROS (62) by regulating antioxidant reactions in the applied plants, and it resolves ROS through the antioxidant mechanism to protect crops from oxidative damage. Zhou et al. (63) found Se reduced CD-bioaccumulation and MDA levels and enhanced the antioxidant activity, growth, and grain yield of supplemental wheat plants. Plants are important carriers of selenium cycling in nature. Inorganic selenium in the soil is transformed into organic selenium through absorption and assimilation by plants. Subsequently, it is taken up by humans and animals and transformed into selenase, selenoprotein, selenocysteine, and other substances in the body. These compounds participate in important metabolic activities. However, the absorption and transformation of SeNPs by crops have not been well studied.

The optimal selenium treatment of crops can effectively regulate the absorption, transportation, and accumulation of nutrients, as well as enhance the antioxidant capacity and improve the efficiency of photosynthesis. Research shows that SeNPs can improve the yield and quality of agricultural products, and improve the biological safety characteristics of crops and human health. Therefore, SeNPs is particularly important for comprehensive crop research because it is less toxic and better absorbed than inorganic and organic forms. Additionally, it can regulate the growth and metabolism of plants. Different from animals, crops can obtain selenium directly from the environment. Therefore, it is of great significance to clarify the absorption and transformation mechanism of selenium in crops for scientific selenium supplementation.

### 3.1. Regulate crop growth

SeNPs regulates the growth of crops by increasing the flavonoids and other nutrients in crops, but its mechanism is not clear. Li et al. (64) found that leaf application of SeNPs could significantly increase the total antioxidant capacity, total flavonoids, total phenols, and vitamin C levels of celery leaves by 46.7, 50.0, 21.4, and 26.7%, respectively. SeNPs treatment promoted the biosynthesis of flavonoids, such as apigenin (58.4%), rutin (66.2%), para-coumaric acid (80.4%), ferulic acid (68.2%), luteolin (87.0%), and kaolin (105.7%). The content of nutrients, such as arginine, tryptophan, and beta-carotene, also increased by 147.8, 91.5, and 61.4%, respectively. Among the other components, the contents of α-linolenic acid (73.8%), 13(S)-hydroxylinolenic acid (65.0%), 12-oxydienoic acid (197.5%), and jasmonic acid (72.9%) were increased. The results indicated that SeNPs could stimulate the level of nutrients in celery by regulating the α-linolenic acid pathway to improve the antioxidant capacity of celery. SeNPs can affect the synthesis of flavonoids and improve the antioxidant capacity through the α-linolenic acid pathway, thus improving the nutrient composition of crops. Rice production under salt stress continues to be a major challenge in many arid countries, and the use of SeNPs may be an effective way to solve the increasingly serious soil salinity problem. Badawy et al. (65) studied the effects of soaking and foliar application of NPs-Se on short root characteristics, physiological traits, and yield of two rice varieties grown in saline soil. The results showed NPs-Se could reduce the effects of harmful salinity, promote crop growth, and increase grain yield.

### 3.2. Regulate crop physiological metabolism

The application of selenium nanoparticles (SeNPs) via foliage spraying or soil irrigation proved to be effective under abiotic stress, which reprogrammed the crop metabolome to a large extent, to reduce the abiotic stress between SeNP and the signal pathway (66). Through the study of celery, it was found that page application of SeNPs could significantly improve the total antioxidant capacity, total flavonoids, total phenols and vitamin C levels of celery leaves by 46.7, 50.0, 21.4, and 26.7%, respectively.

Azimi et al. (67) discovered a combined nano-form of selenium and CS (Cs-Senp). Using CS as a selenium carrier and a controlled release agent, the efficiency of selenium can be improved, especially under low dose stress conditions. To test this, a combination of selenium (10 mg L−1), CS (0.1%) and Cs-SE-NPs (at two concentrations: 5 and 10 mg L−1) were applied to a Moldavian plant at the dosage of 0, 2.5 and 5 mg/kg. Most of the Se and CS-Se-NPs treatments were able to reduce MDA and H_2_O_2_ by improving agronomic traits, photosynthetic pigments, chlorophyll fluorescence parameters, activities of SPAD, proline, phenols, antioxidant enzymes and some main components of essential oil, and alleviating the negative effects of Cd stress conditions.

Zeinali et al. (68) studied and determined the effect of soil selenium treatment (0, 1, 5, and 10 mg/kg) on selenium bioenrichment in oat grains, compared with bulk element selenium or selenate (Na_2_SeO_4_). The soil SeNP treatment significantly increased the selenium concentration in oat grains from 1 to 10 mg/kg (*p* < 0.05). The distribution of selenium accumulated in oat tissues followed the descending order of root and grain > shell > stem and leaf. Soil selenate treatment with 5–10 mg/kg reduced grain yield, while soil SeNP treatment with 1–10 mg/kg significantly increased grain yield, compared to the control. The concentration of selenium in oat grains treated with SeNP was about 7–20 times higher than that treated with soil bulk element selenium, but it was about 7–26% of that in oat grains treated with soil selenium. Therefore, nano-scale elemental selenium particles can be used to develop soil Se-modified fertilizer for selenium-biofortified oat.

### 3.3. Regulate crop gene expression

Selenium is absorbed and transported in crops in a way similar to sulfate and phosphate. It can replace sulfur at the cellular level to synthesize important macromolecules, including amino acids, specific structural and functional proteins and/or potentially toxic non-specific proteins, and other selenium compounds (69). Behbahani et al. (70) studied the benefits and toxicity of SeNPs on balsam pear seedlings, and the application of SeNPs at different stages of plant cell and tissue culture altered cell division and tissue differentiation, epigenetics, transcriptional profiling, and metabolic methods.

Li et al. (71) used transcriptomics and target metabolites to determine differential metabolites and plant signal transduction and lignin biosynthesis genes. The Cd1Se0.2 treatment significantly increased the number of lignin-related genes (PAL, CAD, 4CL, and COMT), as well as the contents of metabolites (ersinol, phenylalanine, p-coumarin, cafestol, and coniferaldehyde). Thus, the integrity of root cell walls was maintained. It also enhanced signal transduction and response resistance of plant hormones by inducing the biosynthesis of genes (BZR1, LOX3, and NCDE1) and metabolites (brassinosteroids, abscisic acid, and jasmonic acid) in roots and leaves. Li et al. (72) studied the effects of Cd contamination soil stress and SeNPs (1, 5 and 20 mg/L) on plant metabolism, fruit nutritional quality, and the composition of volatile organic compounds (VOCs) in pepper. SeNPs (5 mg/L) induced the expression of phenylpropane branch-chain fatty acid pathway (BCAT, Fat, AT3, HCT, and Kas) genes and increased the levels of capsaicin (29.6%), nordihydrocapsaicin (44.2%), and dihydrocapsaicin (45.3%). The concentrations of VOCs (amyl alcohol, linalool oxide, e-2-heptanal, 2-hexenal, ethyl crotonate and 2-butanone) and SeNPs, related to crop resistance and quality, increased significantly. Therefore, SeNPs improved the health of capsaicin crops by regulating the metabolic pathway of capsaicin and the content of amino acids and VOCs.

### 3.4. Regulation of crop growth via the soil micro-ecology

Elemental selenium is one of the main selenium species in aerobic and anaerobic soil, making up 26–66% of the total selenium in soil (73). Gray Selenium and Black Selenium are not biologically active, possibly due to their insolubility, while Red SeNPs have scavenging effects on various free radicals (74, 75). Li et al. (76) used metabolomics to study the rhizosphere soil and pepper plant microbial diversity and the correlation between environmental variables, microorganisms, metabolic pathways, and Se and Cd forms under the intervention of SeNPs. The authors found microbial community changes were closely related to environmental indices, enzymes, soil metabolites, and Se forms. The Cd bioavailability and Cd accumulation in pepper crops decreased. SeNPs application integrated soil–plant balance and enhanced crop defense by improving soil quality and distributing signal molecule levels in rhizosphere soil and pepper crops. Moreover, the level of soil and plant signaling molecules improved after SeNPs reinforcement, and soil enzymes, metabolites (luciferin diacetate, urease, brassinolactone and p-hydroxybenzoic acid), and crop metabolites (rutin, luteolin, brassinolactone, and abscisic acid) were enhanced. The bioenhancement of selenium nanoparticles can promote the growth of beneficial microorganisms, such as gamma-proteobacteria, α-proteobacteria, *Bacteroides*, and anaerobic microbes, in rhizosphere soil (77–79).

Liu et al. (80) investigated the transformation of exogenous SeNPs and selenite (SeO_3_^2−^) in soil and discussed their effects on soil microbial communities and typical microorganisms. Seleomonas had a slow-release effect in soil, promoting the growth of soil microorganisms and enriching soil probiotics. SeO_3_^2−^ is transformed into a stable and low-toxic state in the soil, which increases the content of free radicals and reduces the abundance and diversity of microorganisms. The impact of SeNPs and SeO_3_^2−^ on two typical soil microorganisms (*Bacillus* sp. and *Escherichia coli*) was also evaluated (81). SeNPs did not readily enter microorganisms, thus demonstrating lower toxicity and higher safety. SeNPs are considered a more environmentally-friendly Se additive for agricultural applications. This work provides useful information for better understanding the environmental fate and behavior of selenium fertilizer in the soil.

### 3.5. Selenium nanoparticles enhanced crop stress tolerance

The naked SeNPs are prone to agglomeration, which causes its particle size to increase to the micron level and thus lose its nano-effect, resulting in a decrease in the biological activity of selenium nanoparticles. Therefore, macromolecular substances (polysaccharides, proteins) are added as templates in the process of preparing selenium nanoparticles to form a template-nano-selenium complex, which can enhance the stability of SeNPs. Some studies have shown selenium has a positive impact on crop growth and abiotic stress tolerance at relatively low concentrations (82). In recent years, the potential role of selenium in reducing the toxicity of heavy metals (including Cd) in crops has been of primary interest. SeNPs bio-enhancement mitigates Cd stress by reducing the level of Cd in crop tissues and promoting the accumulation of biomass.

To determine the effect of SeNPs on tomato growth, antioxidant response, and fruit quality under NaCl stress, Espinoza et al. (83) used four doses of SeNPs (1, 5, 10, and 20 mg L^−1^). The application of SeNPs reduced the effect of salinity on the growth of tomato crops. In addition, it had a positive impact on the content of certain biological compounds that are beneficial to human health. However, the content of enzyme and non-enzyme compounds in tomato leaves and fruits increased significantly.

**Table 2:** Effect of SeNPs in human and animals.

Effect	Organisms	References
Promote protein repair, enhance the recovery of antioxidant enzyme activity, reduce lipid metabolism and cell apoptosis, has the effect of anti-heat stress	Cold water rainbow trout	(84)
Improve antioxidant capacity	Tibetan gazelle	(85)
Protect lung injury	Rat	(86)
Improve antioxidant or immune properties to improve growth performance	Broilers under moderate and high temperature	(87)
Relieve bacterial infection and improve inflammation	*Eriocheir sinensis*	(88, 89)
Effectively promote growth, intestinal health, blood health, oxidation status and immune-related gene expression	Nile tilapia	(90, 91)
Anti-inflammatory effect	Irradiated rats	(92)
Maintain optimal growth performance, blood biochemical indices, antioxidant status and immune-related genes	European bass	(93)
Relieve diabetes and kidney disease	Female rats with gestational diabetes	(94)
Improve antioxidant capacity and immune function, reduce oxidative stress	Intrauterine growth retardation piglets	(95)

Liu et al. (96) discussed the repair effect of SeNPs on tomato under penthiopyrad (Pen) stress and selected the optimal concentration and application time to maximize the repair effect without causing phytotoxicity. Pen induced severe oxidative stress in tomato, which inhibited fruit growth and flavor quality. In the immature green stage, 1 mg/L Se-NPs significantly increased the antioxidant capacity of tomato and reduced MDA content. In the 1 mg/L Se-NPs + Pen group, hormone synthesis was normal. The contents of soluble sugars, volatile compounds, and nutrients increased, while the contents of organic acids decreased. Ultimately, the fruit flavor and quality were restored.

## 4. Role of selenium nanoparticles in animals or humans

In addition to enhancing the quality and yield of crops and regulating the resistance of crops, selenium is also a necessary trace element in human and animal life activities (Table 2). It can participate in many physiological metabolic processes in the body. Compared with traditional selenium supplements (inorganic and organic selenium), SeNPs have a higher absorption rate and biological activity. For aquatic organisms, different fish need different amounts of selenium to maintain normal growth, development, and health. The difference in selenium content depends on the characteristics of fish, including species, size, health status, selenium forms (organic and inorganic), and nano forms, as well as environmental conditions, diet, and water quality (97). Selenium also plays an important role in the body’s antioxidant system, immune cell function, growth promotion, sperm formation and migration, and prostaglandin function (98). Jia et al. (99) characterized intestinal flora associated with low and high concentrations of SeNPs in the diet of Chinese tongue sole by 16S rRNAV3-V4 sequencing. The species diversity of low-dose and high-dose SeNPs treatment groups was significantly different from that of the control group. After SeNPs treatment, *Acinetobacter L*, *Arthrobacter* spp., and *Streptophytes* spp. had the highest enrichment degree, and the weight gain rate of the high-dose group was 9% higher than that of the control group.

Abdel-Wareth et al. (100) also discussed the effects of SeNPs, garlic oil, and their combination on the nutrient digestibility, semen quality, serum testosterone, and metabolites of California male rabbits. From the perspective of serum metabolites, the addition of SeNPs, garlic oil, and their combination significantly improved the liver and kidney function and the serum testosterone hormone level. Thus, these treatments were linked to specific health improvements in male rabbits.

### 4.1. Antibacterial effect

SeNPs have significant and sustained bacteriostatic effects on Gram-positive and Gram-negative bacteria, such as *S. aureus*, *S. epidermidis*, *Pseudomonas aeruginosa*, and *E. coli* (101). SeNPs can induce bacteria to produce a large amount of ROS, which destroys the redox balance mechanism in bacteria and causes oxidative damage to the lipids, proteins, and DNA structures of the oxide membrane (102). Zonaro et al. (103) reported that in bacterial biofilms and bacterial suspensions, the amount of ROS induced by SeNPs was higher than that of the selenite group. With the increase in ROS production, the toxicity of SeNPs to bacteria gradually increased. Nguyen et al. (104) studied the bacteriostatic properties of selenium NPs against four foodborne pathogenic bacteria (*E. coli* O157:H7, *S. aureus*, *Salmonella* spp., and *Listeria monocytogenes*). SeNPs proved to be an antibacterial agent that inhibited the growth of *S. aureus* and can be used for food safety applications. Some studies have shown the antibacterial mechanism of SeNPs may include the production of ROS with protein dysfunction, DNA damage, and the disruption of cell integrity to stop bacterial growth (105).

In addition, Geoffrion et al. (30) reported a dose-dependent antibacterial effect of SeNPs on the standard and antibiotic resistance phenotype of Gram-negative and Gram-positive bacteria in the concentration range of 0.05–25 ppm. In addition, SeNPs showed low cytotoxicity when cultured with human skin fibroblasts at concentrations up to 1 ppm, showing an anticancer effect on human melanoma and glioblastoma cells in the same concentration range.

Cremonini et al. (106) found SeNPs effectively degraded the extracellular polysaccharide matrix of *P. aeruginosa*, thereby inhibiting biofilm formation. When the concentration of SeNPs was 50–100 μ At g/mL, the inhibition rate was as high as 70–90%, and the degradation rate was 50–70%. Biofilms are highly organized membrane structures formed by bacteria through the secretion of extracellular polymer. Biofilms can resist antibacterial drugs and fungicides. At the same time, the bacteria in the biofilm are often in a dormant state, with inactive metabolism and strong tolerance to adverse environmental conditions (107). In addition, the porous antibacterial collagen scaffold synthesized by Dorazilova et al. (108) containing chitosan and SeNPs as antibacterial agents, showed good resistance to drug-resistant bacteria. When the concentration was 10 mg/L, its inhibition rate to drug-resistant strains could reach 85%. SeNPscan also effectively prevent and remove biofilms related to bacterial pathogenicity and drug resistance.

### 4.2. Antioxidant effect

Compared to organic selenium, nanoscale elemental selenium (SeNPs) has the same efficacy in increasing the activity of glutathione peroxidase and thioredoxin reductase, but with much lower toxicity than the median lethal dose, acute liver injury, and short-term toxicity. Studies have shown that SeNPs can act as antioxidants and reduce the risk of selenium toxicity (109). Selenium enhances the productive performance and antioxidant activity of animals, especially in hot summer environments (110), and both an increase in surface volume ratio and a decrease in particle size of selenium nanoparticles enhance its biological activity, and selenium amino acids, selenomethionine and selenocysteine are involved in many biomedical effects of selenium with important roles in scavenging free radicals and protecting against oxidative stress (109). SeNP has demonstrated efficacy in scavenging free radicals in both *in vitro* and *in vivo* settings, as well as safeguarding DNA against oxidative damage (111). The addition of SeNPs to the diet can enhance the antioxidant capacity and oxidative stability in the body of mice, and the addition of 0.3 to 0.5 mg/kg of selenium nanoparticles is beneficial in improving the antioxidant capacity, and the maximum amount of SeNPs should not exceed 1.0 mg/kg (112).

Zhang et al. (113) used green tea extract as a reducing agent in the environmentally-friendly preparation of *Lycium barbarum* polysaccharide-capped SeNPs at room temperature. The structure, size, and morphology of the nanoparticles were analyzed by various characterization techniques. The functionalized nanoparticles showed high antioxidant activity, including DPPH and ABTS free radical scavenging ability. The results indicate SeNPs could be used as antioxidant food supplements. Chen et al. (114) synthesized chitosan–selenium (Cs-Se) nanocomposites through an innovative method. First, CS was used as a reducing agent and stabilizer to synthesize selenium nanoparticles (SeNPs), and then the temperature was controlled to synthesize well-dispersed Cs-SE nanocomposites through a simple one-pot reaction. The size range of CS-Se nanocomposites was 83–208 nm. The antioxidant activity of CS-Se nanocomposites was investigated by DPPH, ABTS•+, hydroxyl radical, metal ion chelation, and reducing power measurements. The activity of CS-SE nanocomposites was concentration-dependent and size-dependent, with good antioxidant activity. Therefore, CS-Se nanocomposites may be a more suitable form of selenium supplementation to achieve antioxidant goals in food.

SeNPs have potential antioxidant activity, and its toxicity is low because its redox state is zero (Se0). The inhibition of SeNPs on active oxygen is enhanced, indicating SeNPs can improve its ability to scavenge free radicals by increasing the content of total antioxidants. In addition, contact with plants resulted in changes in fatty acid composition and protein characteristics. Therefore, SeNPs can be applied to crops to improve the quality, yield, and biological safety of crops (115).

Shen et al. (85) analyzed the content of mineral elements in soil, feed, and animal tissues. In addition, blood parameters and the antioxidant index of selenium-deficient Tibetan antelope were evaluated to determine the effect of SeNPs on the antioxidant system. SeNPs significantly improved the selenium content in blood, as well as the antioxidant capacity of the selenium-deficient Tibetan antelope.

### 4.3. Anticancer and anti-tumor effects

In addition to the role of selenoproteins in the antioxidant defense system, selenium plays a vital role in the immune system, impaired fertility, cardiovascular diseases, diabetes, inflammatory diseases, and many types of cancer (i.e., gastrointestinal and prostate cancer) (116). Compared with other forms of selenium, SeNPs exhibit significant anticancer effects and is less toxic. The anticancer mechanism of SeNPs is to induce the production of ROS in cells, induce DNA damage, inhibit the cell cycle, and induce mitochondrial dysfunction (117).

The surface modification of SeNPs with polysaccharides, surfactants, biological macromolecules, and ligands can improve the targeting of selenium nanoparticles to cancer cells and, thus, improve its anti-cancer effect. Yang Fang et al. developed a method for functionalizing SeNPs with polysaccharides from spirulite algae (118). Under the optimized conditions, monodisperse and uniform spherical SPS-SeNPs, with diameters ranging from 20 to 50 nm, were achieved. The nanoparticles were stable in solution for at least 3 months, and the cell uptake and anticancer activity of SPS-SeNPs were evaluated. In melanoma A375 cell lines, chromatin concentration, DNA fragmentation, and phosphatidylserine translocation inhibited cell proliferation due to the action of SeNPs. SPS surface modification significantly enhanced SeNPs uptake and the cytotoxicity of several human cancer cell lines.

Tian et al. (119) studied the effects of SeNPs combined with radiotherapy on proliferation, migration, invasion and apoptosis of A549 and NCI-H23 cells in non-small cell lung cancer (NSCLC), and found that SeNPs, as a new form of selenium, combined with radiotherapy. It has anti-cancer activities such as inhibiting proliferation, invasion, migration and promoting apoptosis of NSCLC cells. Liu et al. (120) used the synthesis of folic acid (FA) -coupled SeNPs as a cancer-targeted nanodrug delivery system for Rupyridine (RuPOP) to exhibit strong fluorescence, which allows direct imaging of cellular transport in nanosystems that can effectively combat multidrug resistance in liver cancer. FA-SeNPs showed low acute toxicity *in vivo*, which verified the safety and application potential of FA-SeNPs as nanomaterials. Hualian Wu et al. (121) have shown that nano-selenium complex modified with mushroom polysaccharide protein (MPSP) has high stability and long retention time *in vivo*, which is a good choice for the treatment of breast cancer. It induces apoptosis of breast cancer MCF-7 cells through cleavage of DNA repair enzymes poly ADP-ribose polymerase (PARP) and activation of apoptosis factors caspase-7, 8, 9.

SeNPs have anti-tumor effects alone and in combination with other active ingredients. At safe concentrations, it can be normally absorbed by the human body, and its working range is larger than that of current traditional selenium supplements. In addition, SeNPs can be used as a raw material for the selenium fortification of foods and be directly absorbed by the human body. Nano-selenium is an excellent active molecular carrier, onto which curcumin, fucoidan, and other small molecular substances with anti-tumor activity can be loaded. However, the direct use of SeNPs as a raw material for nutritional fortification remains to be comprehensively and systematically studied.

## 5. Summary and prospect

In nature, selenium primarily exists in the form of selenite (Se^4+^) and selenate (Se^6+^). Inorganic selenium has high cytotoxicity and is difficult to be absorbed by the human body. Therefore, the safe dose range is narrow. However, SeNPs have low toxicity and high biological activity, including antioxidation, anticancer, and immunity enhancement. Owing to its higher safety, selenium nanoparticles have greater potential than inorganic selenium in preventing selenium deficiency diseases. SeNPs have good antibacterial, antioxidant, anticancer, and anti-tumor activities, being widely used in medical and clinical fields. At present, to improve nutrition and overcome malnutrition, people increase the content of micronutrients in the edible part of crops through crop breeding, reduce the ingredients or microbial toxins in food that affect food quality and human safety, and carry out scientific evaluation and develop biofortification strategies with the help of other scientific methods, such as diet matching (diversification) (122). They also use artificial nutrition fortification and other means to improve the diet of nutritionally vulnerable groups. In this paper, the effects of SeNPs on crops and its biological effects were reviewed, providing insights into crops with nano-selenium fortification.

This paper also reviewed recent research on the effect of SeNPs on animals and humans. The development of nano-selenium-fortified functional food is beneficial to the health of all individuals. SeNPs prepared by microorganisms is stable, safe, and has a good absorption effect. Thus, SeNPs is not only an important selenium nutritional supplement but also has great development prospects in the functional food industry. However, due to the aggregation and deactivation of nano-selenium, stabilizers are usually added during the preparation of the SeNPs to improve its stability, regulate its morphology and size, and improve its biological activity. Polysaccharides have good biocompatibility, strong stability, and diverse functions, and they have been successfully used for the stable preparation and functional modification of SeNPs.

SeNPs is a new material prepared by nanotechnology, with prospects in medicine, nutrition, and agriculture. Nanomaterials and nanotechnology play significant roles in pharmaceutical preparation and clinical practice today. Although the biological synthesis of SeNPs has great potential and broad application prospects, there are still many problems, such as the low tolerance of strain selenium, the difficulty in controlling the particle size, the incomplete conversion rate, the large number of production control factors, selenium loss during production and processing, challenges in large-scale production, and the dose dependence of SeNPs toxicity. Further research is needed to better understand the molecular mechanisms and biological functions of SeNPs.

## Author contributions

QL and TZ: literature collection, manuscript writing, and review. QW, PX, and MQ: data statistics. DT: project management. All authors contributed to the article and approved the submitted version.

## Funding

This work was supported by Key Laboratory of Se-enriched Products Development and Quality Control, Ministry of Agriculture and Rural Affairs/National-Local Joint Engineering Laboratory of Se-enriched Food Development (Se-2021C03) and Sichuan Natural Science Foundation Project (2023NSFSC1229).

## Conflict of interest

The authors declare that the research was conducted in the absence of any commercial or financial relationships that could be construed as a potential conflict of interest.

## Publisher’s note

All claims expressed in this article are solely those of the authors and do not necessarily represent those of their affiliated organizations, or those of the publisher, the editors and the reviewers. Any product that may be evaluated in this article, or claim that may be made by its manufacturer, is not guaranteed or endorsed by the publisher.
